# *Escherichia coli* CRISPR arrays from early life fecal samples preferentially target prophages

**DOI:** 10.1093/ismejo/wrae005

**Published:** 2024-01-14

**Authors:** Moïra B Dion, Shiraz A Shah, Ling Deng, Jonathan Thorsen, Jakob Stokholm, Karen A Krogfelt, Susanne Schjørring, Philippe Horvath, Antoine Allard, Dennis S Nielsen, Marie-Agnès Petit, Sylvain Moineau

**Affiliations:** Département de biochimie, de microbiologie, et de bio-informatique, Faculté des sciences et de génie, Université Laval, Québec, QC G1V 0A6, Canada; Groupe de recherche en écologie buccale, Faculté de médecine dentaire, Université Laval, Québec, QC G1V 0A6, Canada; Copenhagen Prospective Studies on Asthma in Childhood, Herlev and Gentofte Hospital, University of Copenhagen, Ledreborg Alle 34, 2820 Gentofte, Denmark; Food Science, University of Copenhagen, Rolighedsvej 26, 1958 Frederiksberg, Denmark; Copenhagen Prospective Studies on Asthma in Childhood, Herlev and Gentofte Hospital, University of Copenhagen, Ledreborg Alle 34, 2820 Gentofte, Denmark; Novo Nordisk Foundation Center for Basic Metabolic Research, Faculty of Health and Medical Sciences, University of Copenhagen, Blegdamsvej 3B, 2200 Copenhagen, Denmark; Copenhagen Prospective Studies on Asthma in Childhood, Herlev and Gentofte Hospital, University of Copenhagen, Ledreborg Alle 34, 2820 Gentofte, Denmark; Food Science, University of Copenhagen, Rolighedsvej 26, 1958 Frederiksberg, Denmark; Department of Bacteria, Parasites and Fungi, Statens Serum Institut, Artillerivej 5, 2300S Copenhagen, Denmark; Department of Science and Environment, Roskilde University, Universitetsvej 1, 4000 Roskilde, Denmark; Department of Bacteria, Parasites and Fungi, Statens Serum Institut, Artillerivej 5, 2300S Copenhagen, Denmark; IFF Danisco, Health & Biosciences, Dangé-Saint-Romain 86220, France; Département de physique, de génie physique et d’optique, Université Laval, Québec, QC G1V 0A6, Canada; Centre interdisciplinaire en modélisation mathématique, Université Laval, Québec, QC G1V 0A6, Canada; Food Science, University of Copenhagen, Rolighedsvej 26, 1958 Frederiksberg, Denmark; Université Paris-Saclay, INRAE, AgroParisTech, Institut Micalis, Jouy-en-Josas 78350, France; Département de biochimie, de microbiologie, et de bio-informatique, Faculté des sciences et de génie, Université Laval, Québec, QC G1V 0A6, Canada; Groupe de recherche en écologie buccale, Faculté de médecine dentaire, Université Laval, Québec, QC G1V 0A6, Canada; Félix d’Hérelle Reference Center for Bacterial Viruses, Faculté de médecine dentaire, Université Laval, Québec, QC G1V 0A6, Canada

**Keywords:** CRISPR, phage, bacteriophage, E. coli, virome, gut, microbiome, phage resistance

## Abstract

CRISPR–Cas systems are defense mechanisms against phages and other nucleic acids that invade bacteria and archaea. In *Escherichia coli*, it is generally accepted that CRISPR–Cas systems are inactive in laboratory conditions due to a transcriptional repressor. In natural isolates, it has been shown that CRISPR arrays remain stable over the years and that most spacer targets (protospacers) remain unknown. Here, we re-examine CRISPR arrays in natural *E. coli* isolates and investigate viral and bacterial genomes for spacer targets using a bioinformatics approach coupled to a unique biological dataset. We first sequenced the CRISPR1 array of 1769 *E. coli* isolates from the fecal samples of 639 children obtained during their first year of life. We built a network with edges between isolates that reflect the number of shared spacers. The isolates grouped into 34 modules. A search for matching spacers in bacterial genomes showed that *E. coli* spacers almost exclusively target prophages. While we found instances of self-targeting spacers, those involving a prophage and a spacer within the same bacterial genome were rare. The extensive search for matching spacers also expanded the library of known *E. coli* protospacers to 60%. Altogether, these results favor the concept that *E. coli*’s CRISPR–Cas is an antiprophage system and highlight the importance of reconsidering the criteria use to deem CRISPR–Cas systems active.

## Introduction

Clustered Regularly Interspaced Short Palindromic Repeats (CRISPR–Cas) systems are defense mechanisms found in numerous bacterial and archaeal genomes. Cells carrying active CRISPR–Cas systems are protected against phages, plasmids, and other invasive genetic materials. The system is based on recognizing and then cutting of foreign genetic sequences [1, 2]. This occurs when spacers, which are short sequences present in the CRISPR array, are identical to regions (called protospacers) in the invading genetic material. Upon infection, transcribed CRISPR arrays guide Cas nuclease proteins toward protospacer sites in the phage or plasmid genome for DNA or RNA cleavage, preventing infection and making the host cell resistant [2, 3]. The structure of the CRISPR array is usually adaptive and dynamic because new spacers can be acquired over time (mostly at the 5′ leader end of the array) or native spacers can be deleted.

CRISPR–Cas systems are highly diverse in terms of organization, prevalence, and activity. They are currently organized into two classes, six types and several subtypes based on the architecture of the genomic loci and the composition of *cas* genes [4]. Because prokaryotes carry several additional defense mechanisms, their reliance on CRISPR–Cas to evolve phage resistance varies depending on environmental conditions. It has been reported that the presence of CRISPR in microbes correlates negatively with the oxygen level and positively with temperature [5]. Other biotic factors that can be used to predict CRISPR prevalence in microbial ecosystems include viral abundance and diversity [6]. For example, in mixed culture, *Pseudomonas aeruginosa* preferentially uses CRISPR–Cas to defend against phages, while in pure culture, phage receptor mutants are favored [7]. When this bacterial species encounters bacteriostatic antibiotics, the proportion of CRISPR-mediated resistant cells also increases [8].

*Escherichia coli* strains may harbor multiple CRISPR arrays within their genomes, and analyses of the associated *cas* genes indicated that these CRISPR–Cas systems belong to Subtypes I–E or I–F [9]. Both subtypes are rarely found in the same genome [10], and most *E. coli* strains only have Types I–E CRISPR–Cas system. Unfortunately, the nomenclature for *E. coli* CRISPR arrays is inconsistent throughout the literature. There are two arrays associated with the Types I–E system: CRISPR1 (or CRISPR 2.1), located downstream of the *iap* gene, and CRISPR2 (or CRISPR 2.2 and CRISPR 2.3), located between genes *ygcE* and *ygcF* [11, 12]. In some strains, a 0.5-kb AT-rich sequence splits the CRISPR2 array, hence the alternative CRISPR 2.2–2.3 nomenclature. CRISPR1 and CRISPR2 are separated by ~20 kb and only CRISPR1 has associated *cas* genes. There are also two arrays associated with the Types I–F system, designated as CRISPR3 (or CRISPR 4.1) and CRISPR4 (or CRISPR 4.2). However, they are not as well studied, but they are less diverse and less prevalent than CRISPR1 and CRISPR2.

In the *E. coli* K12 laboratory strain, the Types I–E CRISPR–Cas system is repressed by the histone-like nucleoid-structuring protein [13], which is a global transcriptional repressor. The system can, however, be made active by genetically engineering it to efficiently acquire new spacers as well as block phage infection and plasmid transformation through DNA interference [3, 14]. In natural isolates, the activity of the system has not been demonstrated [11]. Although the spacers are highly diverse, some *E. coli* CRISPR arrays have been shown to even remain stable for 42 000 years [15], suggesting that the system rarely acquires new spacers. In fact, it appears that most of the CRISPR diversity in strains of the serotype *E. coli* O157:H7 is driven by spacer deletion rather than acquisition [16]. However, there is a negative correlation between the presence of CRISPR arrays and the pathogenic potential of *E. coli* strains [10]. Given the role that CRISPR–Cas systems play in immunity, an active system would likely prevent the acquisition of new genes coding for virulence factors via horizontal gene transfer. Thus, some environmental conditions might favor the activity of the CRISPR–Cas system in *E. coli*.

Here, we evaluated the CRISPR diversity in *E. coli* isolates that originate from a large collection of fecal samples from children under 1 year old. Using also the viral and bacterial metagenomes from the same samples and reference databases, we found targets for 60% of the spacers, significantly expanding the library of known targets and revealing that *E. coli* spacers preferentially target prophages. Combined with rare events of prophage-targeting spacers being present in the same genome as their targets, these results suggest that *E. coli* CRISPR1, despite a low activity of spacers acquisition, exhibits an antiprophage interference *in vivo*.

## Materials and methods

### Bacterial strains

*Escherichia coli* isolates were isolated from the fecal samples of 648 children enrolled in the Copenhagen Prospective Studies on Asthma in Childhood 2010 (COPSAC2010) mother–child cohort [17]. Fecal samples were diluted and plated in aerobic, microaerophilic, and anaerobic conditions on nonselective and selective media [18]. Bacterial identification was confirmed biochemically, as described previously [18]. Altogether, 348, 467, and 954 *E. coli* isolates were obtained from fecal samples obtained 1 week, 1 month, and 1 year after birth, respectively, from 639 children. The 1769 *E. coli* isolates were stored at −80°C in 20% glycerol at the Statens Serum Institut (Copenhagen, Denmark) in 96-well plates.

### PCR and Sanger sequencing

Isolates were first transferred in fresh TSB medium in 96-well plates and were incubated at 37°C overnight. To screen for the CRISPR1 locus of the *E. coli* strains, primers 5’-GATGGGTTTGAAAATGGGAGCTGGG-3′ and 5’-AGACGTATTCCGGTGGATTTGGATGG-3′ were used. These primers anneal the *iap* and *cas2* genes, respectively. PCR amplification was performed with the *Taq* polymerase (Bio Basic) using the following program: 2 min at 95°C, followed by 35 cycles at 95°C for 20 s, 58°C for 40 s, 72°C for 2 min, and then 1 cycle at 72°C for 5 min. To estimate the amplicon size, 5 μl of PCR product was migrated on a 2% agarose gel. PCR products were then sent for Sanger sequencing (CHUL sequencing platform, Quebec City) with the same primers.

### Assembly and CRISPR identification

Forward and reverse nucleotide sequences were assembled using Geneious v11.1.5 and the De Novo Assemble tool. When sequences failed to assemble or the sequence quality was poor, custom internal primers (Supplementary Table S1) were designed to perform PCR and Sanger sequencing using the same program. Assembled sequences were then exported in FASTA format. CRISPR arrays were identified with CRISPRDetect v2.2 [19], using default parameters, except for the -array_quality_score_cutoff, which was set to 3.

### Bioinformatics analyses

Bioinformatics analyses were conducted in a Jupyter notebook using Python3 packages and software mentioned in Supplementary Table S2. CRISPRStudio [20] was used to produce a color-coded figure of the CRISPR array, with default parameters. Given the size of the dataset, the full color-coded figure was neither practical nor informative as a main text figure. Instead, we used a network representation to illustrate the diversity of spacers and the interconnectivity of the CRISPR arrays. First, a graph was created by identifying each isolate with a node and by linking any two isolates if they shared at least one spacer. The magnitude of each connection was measured using three similarity indices: binary Jaccard, weighted Jaccard, and Tanimoto. We compared the similarity index distributions and found they only marginally differed (see Supplementary Fig. S1). We continued our network construction with similarity values from the binary Jaccard index. The same steps were performed on 500 random datasets, which consisted of spacers randomly distributed among all isolates, with each isolate keeping the number of spacers they had in the original dataset. We looked at the distribution of the value of the similarity indices and compared them to the original dataset and estimated a 95th percentile, i.e. a value under which 95% of the similarity index measured in the random datasets are found. This estimation provided a threshold (0.12) above which any connection in the original dataset were considered as significant (see Supplementary Fig. S2). We then removed any connection with a similarity index below 0.12 to build the final network. The Infomap [21] algorithm was used to extract modules. Cytoscape v3.9.0 was used to edit the network and color the modules.

To distinguish known spacers from new ones, we compared spacers with the CRISPR Spacer database [22] using blastn v2.9.0 [23]. Spacers were considered to be known when they matched (0 mismatch, 100% coverage) a spacer in the database; otherwise, they were considered to be new. Several databases and datasets were queried for the presence of spacer targets (protospacers). These include the NCBI Virus database (filtered for Virus; bacteriophage, Nucleotide completeness; complete, downloaded on 19 April 2021), COPSAC viromes from 1-year fecal samples [24], 46 COPSAC coliphages [25], metagenome-assembled genomes (MAGs) from COPSAC metagenomes from 1-year fecal samples [26], and the NCBI nt database for bacterial genomes (downloaded on 8 October 2021). To determine homology for results from the NCBI Virus database, COPSAC viromes, and COPSAC coliphages, both fasta36 and blastn were performed. For the bacterial genomes and COPSAC metagenomes, only blastn was used. A sequence was considered as a target when the alignment showed at most four mismatches on the full length of the 32 nucleotide-long spacer and zero gap. To avoid misidentifying spacers in CRISPR arrays as targets in COPSAC metagenomes and bacterial genomes, we removed all hits that were within 100 bp of the *E. coli-*conserved CRISPR repeat sequence “5”-CGGTTTATCCCCGCTGGCGCGGGGAACAC-3′.

The presence of prophages within the regions targeted by spacers in the bacterial genomes and COPSAC metagenomes were determined using a three-step approach. First, for bacterial genomes, we determined the gene function of every gene targeted by a spacer using the efetch() function from the Bio.Entrez Python package. Second, for each bacterial genome and COPSAC metagenome targeted by at least two spacers, the median absolute deviation (MAD) was measured. The MAD was calculated according to the position of all the spacer targets on the genome. Third, using only bacterial genomes and COPSAC metagenomes with at least 10 spacer targets, we extracted the putative prophage regions by identifying the minimum and maximum genomic positions of the spacer targets and adding 25 kb both upstream and downstream of the targets. The nucleotides of these putative prophages were pairwise compared with the phages from the NCBI Virus database, COPSAC viromes, and COPSAC coliphages with at least one spacer target using blastn to deduplicate identical sequences. Sequences were deemed to be identical when they shared 95% identity over 85% coverage. Identical sequences were then clustered using MCL [27]. One sequence per cluster was kept as a representative for the following steps. VIBRANT v1.2.1 [28] was used to confirm viral identity and type (virulent/temperate) using the default parameters, except with the -virome argument on. The viral type was manually confirmed by searching for the presence of temperate markers (mainly the serine recombinase, which is overlooked by VIBRANT). The network representation of confirmed prophages and phages were generated with the NetworkX Python package and edited in Cytoscape v3.9.0. The genome map of a representative prophage was done with the VIBRANT annotations, confirmed with the annotations of the bacterial genome on NCBI and visualized with EasyFig v2.2.5 [29].

To study the prevalence of self-targeting events, we selected *E. coli*-complete genomes with at least one CRISPR locus (*n* = 1014) from NCBI. Their corresponding spacers and repeats were extracted from the CRISPR Spacers database. Homology searches between the spacers and the genomes as well as between the repeats and the genomes were performed using blastn. The presence of spacers, both in the CRISPR array (according to the CRISPR Spacers database and if they were within 100 bp of a repeat sequence) and elsewhere in the genome, were considered to be indicative of a self-targeting event. Some genome files contained plasmid sequences. When a spacer was present only in the CRISPR array of the bacterial chromosome and in the plasmid, but not elsewhere, it was not considered to be a self-targeting event. Self-targeting events were identified in a total of 99 genomes (out of 1014, 9.8%). To verify that the self-targeting spacer targeted a prophage within the same chromosome, we checked for the presence of prophages in the 99 genomes using PHASTER [30] and compared the prophage positions with those of the self-targeting events.

## Results

**Figure 1:**
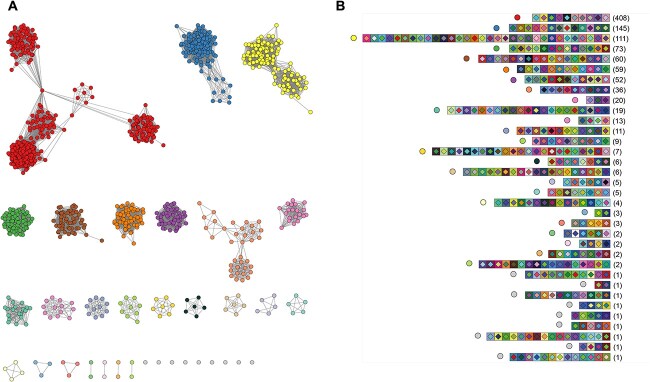
CRISPR diversity in *E. coli* isolates; (A) each node represents a CRISPR array (isolate) and each edge represents a level of shared spacers between two nodes; the length of the edge is indicative of the Jaccard similarity index; the color of the node represents the module the CRISPR array belongs to; (B) one random CRISPR array per module is illustrated; each colored square corresponds to a spacer; the colored dot next to the array refers to the module it represents; two spacers with the same diamond–square color combination share homologous sequences; the numbers in parentheses correspond to the number of CRISPR arrays in each module.

Using PCR, we amplified and then Sanger-sequenced the CRISPR1 loci of 1769 *E. coli* isolates obtained from 639 fecal samples from children enrolled in the COPSAC2010 study. A CRISPR1 array was successfully sequenced for 1048 (59%) isolates, with arrays containing 2–32 spacers (average = 11 spacers). Then, we sought to evaluate the diversity within CRISPR arrays (spacer content) and the interconnectivity of the isolates. Traditional phylogenetic trees are poorly suited for CRISPR arrays because the sequence lengths are highly variable (due to the different numbers of spacers) and this region is prone to recombination events. The alternative approach of representing CRISPR arrays with a color-coded figure was impractical, given the size of the dataset. Thus, we used a network-based approach to visualize the diversity of CRISPR arrays (Fig. 1). For each pair of CRISPR arrays (each CRISPR array is an isolate), a Jaccard similarity index was calculated based on the number of shared spacers, which was then used to generate a network, as shown in Fig. 1A. To group CRISPR arrays into modules, we used Infomap [21], which allowed arrays with slightly different spacer content to still cluster together. This resulted in 34 delimited CRISPR modules. Nine of them were singletons. The remaining 25 modules contained 2–408 CRISPR arrays (Fig. 1B). To appreciate the diversity of CRISPR arrays, we illustrated the spacer content of one randomly selected representative for each module (Fig. 1B).

**Figure 2:**
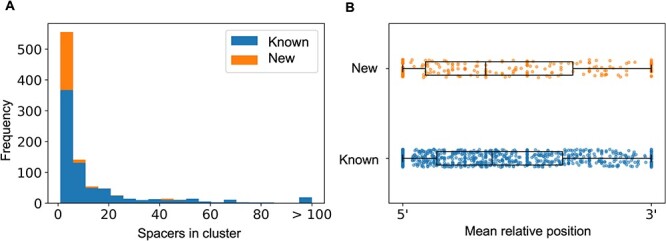
Specificity of the spacers to the early life gut environment; (A) distribution of the number of spacers per cluster, showing known (blue, identical to previously sampled spacers) and new (orange, unique to our dataset) clusters; (B) mean relative spacer position for each spacer cluster along the 5′–3′ axis, grouped according to known (blue, bottom) and new (top, orange) clusters.

To explore the CRISPR diversity further, we studied the spacers in the *E. coli* isolates. A total of 12 298 spacers were extracted from all 1048 CRISPR1 arrays. These spacers corresponded to 946 unique sequences (referred to as spacer clusters) based on sequence homology. Spacer clusters contained 1–279 spacers. We were particularly interested in the specificity of the spacers, such as whether they are specific to the early life gut environment as well as the identity of their targets. We probed several databases for sequence homology. First, we compared our 946 spacer clusters with known spacers found in *E. coli* and in the NCBI database. Only 22% (*n* = 210) of the spacer clusters were considered to be new (no homology with previously sequenced spacers), whereas 78% (*n* = 736) of the spacer clusters were already sampled in published *E. coli* genomes. Spacer clusters with fewer spacers more often included new spacers: the 210 new spacer clusters only comprised 631/12 298 spacers, 5% of the total spacer dataset (Fig. 2A). As new spacer acquisition is mostly polarized at the 5′ end of the CRISPR array, we compared the mean relative position along the 5′–3’ CRISPR array axis of known and new spacers (Fig. 2B). We found no significant difference (Kolmogorov–Smirnov two-sample test, *P*-value = 0.1076) as new and rare spacers were not concentrated at the 5′ end of the CRISPR array. Since new spacers do not appear to have been acquired recently, we concluded that they are not specific to the environment sampled in this study and rather represent rare *E. coli* spacers that had yet to be sampled.

To identify targets (protospacers), we first searched for homologies with phage genomes from three sources: (i) all NCBI phage genomes, (ii) 1-year viral metagenomes obtained from the same children as our isolates, and (iii) a collection of sequenced coliphages amplified from the isolate supernatants [25]. Spacers rarely matched sequences from viral sources. There were 34 spacer clusters that matched 93 phages that infect mostly *Salmonella* and *Escherichia* (see Supplementary Table S3). The most frequently targeted known phages were Escherichia phage P1, Escherichia phage RCS47 and Salmonella phage SJ46 with seven related-protospacers. There were 31 spacer clusters that matched 37 viral contigs from viral metagenomes. In a previous study [24], we performed a host prediction for these viral contigs. *Enterobacteriaceae* was predicted for 14/37 (38%) viral contigs and there was no matching prediction for the others. Lastly, only three spacer clusters targeted three coliphages previously isolated from the same fecal samples (Escherichia phage Evi, LR597642.1; Escherichia phage ESSI2_ev239, NC_049392.1, and Escherichia phage mEp460_ev081, LR597641.1). In total, 133 phage protospacers matched the 74 spacers.

We next explored the NCBI bacterial genomes as well as bulk 1-year metagenomes from the same children as our isolates. Surprisingly, most of these unknown targets could be identified by searching for spacer matches in bacterial genomes. After removing any matches with spacers in CRISPR arrays, 474 spacer clusters were found to target 9321 bacterial genomes from the NCBI database. Roughly half were *E. coli* genomes and the rest were genomes from members of the *Enterobacteriaceae* family (*Klebsiella*, *n* = 2031; *Salmonella*, *n* = 1320; *Enterobacter*, *n* = 365; *Citrobacter*, *n* = 265; *Shigella*, *n* = 202). Of note, the most targeted genome was *E. coli* strain RHB42-C16 (CP056933.1), with spacers targeting 87 different regions (protospacers). We also identified numerous spacer targets in MAGs from the COPSAC 1-year metagenomes: 256 spacer clusters targeted 1702 bacterial contigs. Combined with spacers targeting sequences of viral sources, this reduced the percentage of unknown targets from 95% to 41% (392/946). This apparent preference for targets in prophages could be an artifact of the more exhaustive sequence availability for bacterial genomes, where spacer targets in prophages are identified, compared to phage genomes. Bacterial genomes are >10 times larger and are more frequently found than phage genomes on NCBI. Still, we find the same inclination for temperate phages when we analyzed spacers targeting NCBI phages only. To evaluate if a bias for temperate phages exists, we compared the percentage of temperate phages on NCBI with the percentage of temperate phages targeted by *E. coli* CRISPR spacers. NCBI phages were chosen for this analysis because their genomes are complete, making it possible to accurately determine their lifestyle (whereas, viral contigs from the virome dataset could be incomplete). Since there may be different percentages depending on the bacterial host, we focused on phages that infect *Salmonella*, *Escherichia*, and Enterobacteria (*n* = 2437). These three hosts represent 80% of the targeted phages’ hosts (see Supplementary Table S3). To avoid any overrepresentation, sequences were deduplicated, which resulted in 1243 genomes. We then ran VIBRANT to predict the lifestyle for each phage. VIBRANT predicted that 1058 and 175 genomes were virulent and temperate, respectively. Thus, temperate phages represent roughly 14% of all phages that infect *Salmonella*, *Escherichia*, and Enterobacteria. In comparison, 100% of the phages targeted by spacers (the subset of phages that infect the same three hosts) were temperate. Despite temperate phages being a minority in the NCBI Virus database, they represent the totality of phages being targeted by *E. coli* CRISPR spacers.

**Figure 3:**
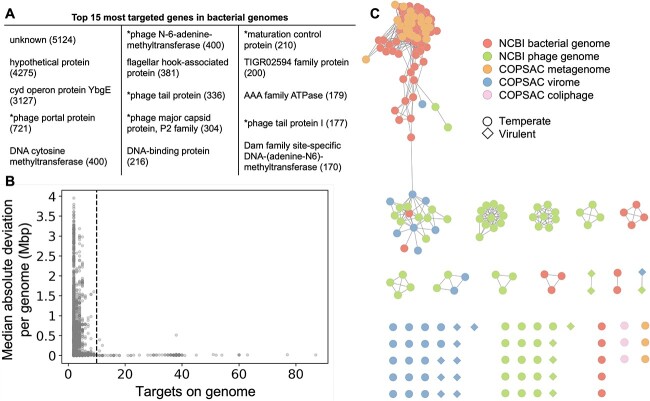
The *E. coli* spacers preferentially target prophages found in *Enterobacteriaceae* genomes; (A) list of the 15 most targeted genes in bacterial genomes; genes marked with an asterisk are strictly phage genes; (B) scatter plot of the number of targets and MAD for each bacterial genome targeted by spacers; the dotted vertical line at *x* = 10 corresponds to the minimum cut-off for bacterial genomes that were investigated for the presence of a prophage; (C) network representation of the viral sequences targeted by spacers; each node is a viral sequence, and each edge between two nodes signifies at least 95% identity over 85% coverage, which is the definition of a viral species; nodes are colored according to their origin (red, prophages in NCBI bacterial genomes; green, NCBI phage genomes; orange, prophage in COPSAC metagenomes; blue, phage in COPSAC virome; and pink, coliphage from the COPSAC *E. Coli* supernatant), and their shapes (circle, temperate; diamond, virulent) are based on their replication mode.

**Figure 4:**
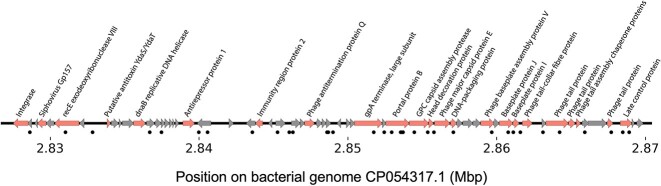
Genome map of a representative prophage of the large cluster in Figure 3; all arrows represent genes; the gray arrows are genes coding for hypothetical proteins or proteins of unknow functions; the black dots under the genome map are regions targeted by spacers, and the numbers are the coordinates of the prophage in the bacterial genome.

We also investigated the targeted genes, and several phage-associated genes were identified to be among the 15 most prevalent (Fig. 3A), such as genes coding for portal proteins, tail proteins, and major capsid proteins. We then examined whether spacers preferentially target prophages in bacterial genomes and MAGs from COPSAC 1-year metagenomes. For each genome, the MAD was calculated using the genomic position of each target. The MAD is a dispersion measure, which tells us about the deviation around the median of a dataset. The smaller the MAD is, the more condensed the data are around its median and conversely, the bigger the MAD is, the more scattered the data are. In this context, the data are the position of each spacer target in a bacterial genome. This metric was used as a proxy to detect prophage regions in bacterial genomes because we hypothesized that genomes with closely located targets (low MAD values) were indicative of a prophage region. The rationale behind this is that if *E. coli*’s CRISPR–Cas system targeted bacterial genomes, we would find spacer targets randomly distributed across the genome, resulting in a high MAD. Conversely, if *E. coli*’s CRISPR–Cas is an antiphage system and we find targets in bacterial genomes, they would be concentrated in specific regions, where prophages are located. This would result in a low MAD. When looking at the relationship between the number of targets and the MAD (Fig. 3B), two distinct groups emerged: genomes with few targets (<10) and high MAD values and genomes with many targets (≥10) and low MAD values (Fig. 3B). A total of 192 genomes (144 *E. coli* genomes, 17 other bacterial genomes, and 31 contigs that belong to 9 MAGs) fell into the latter category and were screened to confirm the presence of a prophage in the region encompassing spacer targets. After analyzing the sequences using VIBRANT, we determined that only 3/192 genomes did not carry a predictable prophage. There was high sequence redundancy because 126 prophages (out of 189) belonged to the same species (Fig. 3C, large cluster at the top left corner, 95% identity over 85% coverage). Together with these 189 prophages, the 133 sequences of viral origin (NCBI phages, COPSAC 1-year viromes, and COPSAC coliphages) were used to evaluate the sequence diversity and determine whether these phages were temperate or virulent (Fig. 3C). The sequence comparison revealed that some viral contigs that were identified in the viral metagenomes were identical to NCBI phages and prophages in bacterial genomes (Fig. 3C, second largest cluster). Overall, we concluded that spacers preferentially target prophages (and temperate phages), as only 18/325 phages were considered to be virulent according to VIBRANT predictions. Genomic organization and annotation of a prophage that is representative of the large cluster are illustrated in Fig. 4 along with the regions targeted by spacers.

The prophage preference of *E. coli* CRISPR arrays prompted us to investigate whether the arrays were targeting resident prophages, or prophages not yet acquired by the cell. We searched for the evidence of self-targeting in *E. coli*, where a spacer and its target are both found in the same genome. We analyzed 1014 complete *E. coli* genomes from NCBI and considered a self-targeting event to have occurred when a spacer sequence was present both in the CRISPR array and elsewhere in the bacterial genome. There were 99 genomes where at least one self-targeting event was identified. That is an occurrence of ~10%. In nine of these genomes, self-targeting spacers targeted both the bacterial chromosome and one or two plasmids. Most importantly, self-targeting spacers directed at a prophage were rare, with only 18 events (out of 99) identified. This result is consistent with a role of *E. coli* CRISPR arrays in preventing the acquisition of new prophages. Another hypothesis that is also consistent with the results is that spacers are left-overs from a warfare with previously resident prophages that have now been excised due to the spacer match. Lastly, we investigated if there were instances where *E. coli* spacers and MAGs or viral contigs originated from the same child. There were four instances where a prophage in a MAG was targeted by a spacer that was found in the same child at the same sampling time (1 year old). We found no viral contigs and spacers that originated from the same child at the same timepoint. These results suggest that *E. coli* spacers rarely match viral invaders that are found in the same gut environment, although a limited sequencing depth could hinder the detection of such an occurrence.

## Discussion

Our objective was to address the question of *E. coli* CRISPR–Cas systems’ activity. To investigate this, we had access to a unique combination of biological data from early life fecal samples (bacterial isolates, bacterial and viral metagenomes, and isolated phages), which we analyzed from a bioinformatics perspective. We evaluated the CRISPR content in *E. coli* isolates originating from fecal samples from children within their first year of life. The percentage (59%) of CRISPR-positive isolates was consistent with isolates from other environments, including human, animal, and water sources [31], suggesting that CRISPR prevalence may not be environment-specific at least for *E. coli*. Next, we used a network-based approach to visualize CRISPR diversity. This allowed us to define CRISPR modules according to spacer content and examine network properties, reflecting the interconnectivity of the isolates.

We then studied *E. coli* CRISPR arrays at the spacer level. The ratio of total spacers to distinct spacer sequences (12 298/946) was consistent with other studies [11, 12, 15, 32]. Two different analyses led to the same conclusion: spacers are not specific to the early life gut environment. First, most of the spacers were identical to those carried by *E. coli* genomes in the NCBI database. Second, there were only rare instances of spacers targeting a viral sequence found in the same sample (same child and timepoint). Again, these results support the hypothesis that the Subtypes I–E CRISPR–Cas system in *E. coli* is inactive because the spacers present in *E. coli* are not tailored to the viral invaders encountered by the bacterial population in its direct environment. This is also consistent with another study, where they recovered *E. coli* spacers from a 42 000-year-old mammoth specimen where some matched with present-day *E. coli* spacers [15].

Our work massively expands the known targets of *E. coli* spacers, revealing that most target prophages. In previous studies, the percentage of spacers matching a target was consistently low, ranging from 0.6% to 12% [11, 12, 15, 16, 31]. Here, we found a target for 60% of the 946 spacer clusters. The vast majority of these targets are found within prophage regions in *Escherichia* chromosomes. It has been previously reported that *E. coli* spacers target prophages [12, 33], but the high prevalence of this occurrence reported in the present study is new. Many targets were identified in bacterial and viral metagenomes from the same cohort. Even though these targets were not sample-specific as already mentioned, these results support the need to combine culturomics and high-throughput sequencing to elucidate spacer targets and to better understand phage–bacteria interactions. We also showed that for phage genomes in the NCBI Virus database, the preference for spacer targets in prophages is not caused by an overrepresentation of temperate phages. Quite the contrary, temperate phages are only a minority in the database but represent the spacer targets for phages that infect *Escherichia*, *Salmonella*, and Enterobacteria. Still, there are methodological and biological reasons that could explain the high prevalence of spacer targets in prophages, and they should be further explored. First, searching for spacer targets in bacterial genomes inevitably increases the potential to find a hit, simply because the bacterial genome database is much larger (more than one million genomes, averaging a few million bp in size) than its phage counterpart (around 30 000 genomes, averaging a hundred thousand bp in size). It would be interesting to examine this with another bacterial species whose CRISPR–Cas system mainly targets virulent phages and to verify whether we also find spacer targets in bacterial genomes. Instead, we argue that this predominance of spacer targets in prophages is the result of specificities in the interactions of *E. coli* and its coliphages. Mathieu and colleagues [25] previously looked at virulent and temperate coliphages originating from the same samples used in this study and found that temperate phages are more prevalent but less infectious than virulent phages. Perhaps the encounter rate is higher for temperate phages, making it more advantageous for *E. coli* to tailor its CRISPR–Cas system against temperate phages.

The high prevalence of spacer targets in prophages is not specific to *E. coli*, as it has been shown for other bacteria, such as *P. aeruginosa* [34], *Flavobacterium columnare* [35], *Paenibacillus larvae* [36], and *Streptococcus pyogenes* [37]. More broadly, a vast bioinformatics analysis led by Shmakov and colleagues [38] showed that, in bacterial and archaeal genomes, nearly all spacers are predicted to match sequences of the mobilome (mobile genetic elements, such as plasmids and prophages). In that same study, self-targeting spacers were nearly absent, which suggested that there is a strong selection against them. This result differs from what we observed in our *E. coli* isolates, as we measured a 10% prevalence of genomes with self-targeting records. The rate of self-targeting events for spacers matching known sequences has not been thoroughly investigated for a broad range of bacteria [39], making it difficult to evaluate if the rate measured in *E. coli* is indicative of an active system or not. However, in *Streptococcus thermophilus*, a model bacterium used for the study of an active Type II-A CRISPR–Cas system, it was found that 7% of the spacers matched the chromosome [40].

The *E. coli* is a fascinating model for the study of the biology of CRISPR–Cas systems because despite being the first organism in which a CRISPR array was identified in 1987 [41], we continue to uncover new aspects of its mechanism. Evidence suggests that the canonical function of its system is inactive: spacer acquisition is not observed in laboratory conditions (due to a well-characterized repressor) and spacers are not specific to invading DNA from the environment (at least not detected using the relatively shallow sequencing depth of many metagenome studies). However, other observations do not support this hypothesis. For example, there is a general mutational bias toward deletion in prokaryotic genomes [42], which makes it difficult to explain how *E. coli* CRISPR arrays that are up to 32 spacers long and from an inactive system could persist and avoid deletion. In addition, we uncovered the targets for 60% of the spacer clusters and found that spacers almost exclusively match prophages. Yet, spacers targeting prophages present in the same bacterial chromosomes are very rare. This could mean that the system effectively protects from temperate phage integration. In *E. coli*, spacers could effectively alter lysogenization, induction, or prophage curing in specific conditions. For example, spacers played a role in protection from a temperate phage (phage lambda) in *E. coli* in laboratory conditions with an overexpressed CRISPR–Cas system [43]. The possibility of CRISPR playing a role in protection is reminiscent of the role of the *P. aeruginosa* CRISPR–Cas system and its interaction with the temperate phage DMS3. This CRISPR–Cas system was first thought to be inactive in protecting against phage infection [34], but it was later found to be active in low nutrient and mixed culture conditions [7, 44]. A CRISPR–Cas system and DMS3 interplay that is not mediated by interference is necessary to alter biofilm formation [45, 46]. This suggests that CRISPR–Cas systems may play other roles beyond their canonical function in temperate phage–bacteria interactions.

Altogether, these results provide a new perspective on the diversity and potential activity of the CRISPR1 system in *E. coli*. With 60% of the spacers matching prophages, we support the idea that its CRISPR–Cas system is an antiprophage system as proposed recently [47]. We hypothesize that studying *E. coli* and temperate phages in diverse conditions, such as in environments that mimic natural ecosystem, may provide additional knowledge about CRISPR-mediated interactions.

## Supplementary Material

Supp_Mat_wrae005

## Data Availability

Spacers from COPSAC *E. coli* strains are available as a supplemental fasta file. Bacterial strains are available upon request.
