# Supplementary material for: Escherichia coli CRISPR arrays from early life fecal samples preferentially target prophages
Source: ISME J. 2024 Jan 14;18(1):wrae005. doi: 10.1093/ismejo/wrae005 (PMC10910852; doi:10.1093/ismejo/wrae005)
Supplement: Supp_Mat_wrae005 [file supp_mat_wrae005.pdf]

Supplementary Table 1: List of primers used in this study

Description	Forward sequence (5'-3')	Reverse sequence (5'-3')
CRISPR1 screening	GATGGGTTTGAAAATGGGAGCTGGG	AGACGTATTCCGGTGGATTTGGATGG
CIP 01D08	GGGTGTTGCAAATATTCTCC	AGATTGCGTCTTGTCGATGG
CIP 01H12	ATACCGCGATTCATGCGACG	CATATAACGAAACGCTGGGG
CIP 02A07	TTTGTGCGGGGAATTATGGG	ATGGAACAGCAATCAGTGCG
CIP 02D07	TTGAAGATCTGGACGCGTGG	TTCAAACAGGCGCGTAATCC
CIP 03A09	AATCACCCCACTGGGTAAAC	CTTTGCTACCCGCTCAAAAC
CIP 03F11	TTCGCGGCGGCATTATTGAG	TTAGGCAGGTGGCGATGTTC
CIP 08D11	TCATTGCCCGTTGAGATACC	TAACGAAACGCTGGGGTATC
CIP 09E09	AGAGAGTGGTTTGAAGCCGC	TTTGCTACCCGCTCAAAACC
CIP 09F01	ATTTCGCGTCAATCTCCACC	TTTGCTACCCGCTCAAAACC
CIP 11H02	GATTAGTCCGGCGTTTCATG	AGGTAGCTGTTATCCGTGTC
CIP 12H08	ATTATCTGACGTGGCTCTCC	AGCCGCTTAAAAGCCAGTTC
CIP 13B01	GATATTCAGGCAGCGTAACG	TTTGCTACCCGCTCAAAACC
CIP 13C01	AACTGGTCAGCAATTCTGGC	GGTAACAATCAGCCACTTGC
CIP 14F11	GTCACGAAACAGACAACACC	GACAGAATATCCGTGTACCC
CIP 15A04	ATTTCGCGTCAATCTCCACC	AGGTAGCTGTTATCCGTGTC
CIP 17C09	GATATTCAGGCAGCGTAACG	GACAGAATATCCGTGTACCC
CIP 17F09	TTCAGTATTGCCGGTGTCAG	GCCAGTTCAATTTGCCATCG
CIP 19C10	ACACGGATAACAGCTACCTG	GACAGAATATCCGTGTACCC

CIP = custom internal primers

Supplementary Table 2: List of software and packages used in this study.

Software/Package	Comment
CRISPRDetect v2.2	-array_quality_score_cutoff 3 because we used fasta files<br>https://github.com/ambarishbiswas/CRISPRDetect_2.2
CRISPRStudio	https://github.com/moineaulab/CRISPRStudio
Cytoscape v3.9.0<br>-clusterMaker app -> MCL	https://cytoscape.org
Fasta36	Command line version can be obtained via conda
MCL v14-137	Command line version can be obtained via conda
CRISPR Spacer database	https://github.com/edzuf/CrisprOpenDB<br>http://crispr.genome.ulaval.ca
Blastn v2.9.0	Command line version can be obtained via conda
VIBRANT v1.2.1	https://github.com/AnantharamanLab/VIBRANT
PHASTER	https://phaster.ca
EasyFig v2.2.5	https://mjsull.github.io/Easyfig/files.html
Jupyter Notebook	
Python v3.7.6	-pandas<br>-matplotlib.pyplot<br>-scipy<br>-collections<br>-networkx<br>-seaborn<br>-Bio.Entrez<br>-sqlite3<br>-random

Supplementary Table 3: Summary of the number of targets for NCBI phages

Phage Name	Number of targets	Accession number
Salmonella virus SJ46	7	KU760857.1
Escherichia virus P1	7	MH445380.1
Escherichia virus P1	7	MH422554.1
Salmonella virus SJ46	7	NC_031129.1
Escherichia virus P1	7	AF234173.1
Escherichia virus P1	7	AF234172.1
Escherichia virus P1	7	NC_005856.1
Escherichia virus RCS47	7	FO818745.1
Escherichia virus RCS47	7	NC_042128.1
Escherichia virus P1	6	AF503408.1
Escherichia virus P1	6	NC_050152.1
Escherichia phage vB_EcoM-Ro157c2YLVW	6	MH160767.1
Escherichia phage vB_EcoM-Ro157c2YLVW	6	NC_050153.1
Escherichia phage CMS-2020a	6	CP053388.1
Escherichia phage CMS-2020a	6	CP054387.1
Escherichia virus P1	5	MH445381.1
Salmonella phage SSU5	2	JQ965645.1
Salmonella phage SSU5	2	NC_018843.1
Bacteriophage sp.	2	MN856003.1
Escherichia phage D6	2	MF356679.1
Escherichia phage D6	2	NC_050154.1
Klebsiella phage vB_Kpn_1825-KPC53	2	CP058330.1
Salmonella phage ST64B	1	AY055382.1
Salmonella phage 118970_sal3	1	KU927493.2
Salmonella phage ST64B	1	NC_004313.1
Salmonella phage 118970_sal3	1	NC_031940.1
Klebsiella phage 5 LV-2017	1	KY271399.1
Edwardsiella phage Edno5	1	MH898687.1
Vibrio phage pYD38-A	1	NC_021534.1
Aeromonas virus pIS4A	1	NC_042037.1
Stx2-converting phage Stx2a_WGPS6	1	AP012539.1
Stx2-converting phage Stx2a_WGPS6	1	NC_049945.1
Escherichia phage GER2	1	MG710528.1
Salmonella phage SW3	1	MK972714.1
Salmonella phage SW5	1	MK972713.1

Salmonella phage SI7	1	MK972712.1
Salmonella phage SI7	1	NC_049460.1
Escherichia phage ESSI2_ev239	1	NC_049392.1
Burkholderia virus ST79	1	KC462197.1
Burkholderia virus ST79	1	NC_021343.1
Vibrio virus Canoe	1	NC_048066.1
Klebsiella phage 2 LV-2017	1	KY271396.1
Siphoviridae sp. ctdc_1	1	MH622927.1
Salmonella phage UPF_BP1	1	KX776161.1
Salmonella phage UPF_BP1	1	NC_047875.1
Aeromonas phage phiARM81ld	1	KT898133.1
Podoviridae sp. ctdb7	1	MH593831.1
Salmonella virus ST64T	1	AY052766.1
Salmonella phage vB_SemP_Emek	1	JQ806763.1
Salmonella phage vB_SalP_PM43	1	MF188997.1
Salmonella virus L cII-101	1	MW013503.1
Salmonella virus L cI-40 13-am43	1	MW013502.1
Salmonella virus ST64T	1	NC_004348.1
Salmonella phage vB_SemP_Emek	1	NC_018275.1
Salmonella phage g341c	1	FJ000341.1
Salmonella phage g341c	1	NC_013059.1
Ralstonia phage Heva	1	MT740742.1
Ralstonia phage Cimandef	1	MT740730.2
Bacteriophage sp.	1	MN855766.1
Burkholderia virus Bcep22	1	AY349011.3
Burkholderia virus Bcep22	1	NC_005262.3
Stx2a-converting phage Stx2_499	1	LC567824.1
Stx2a-converting phage Stx2_12E129_PPompW	1	LC567841.1
Stx2a-converting phage Stx2_14040	1	LC567818.1
Stx2a-converting phage Stx2_14744	1	LC567820.1
Stx2a-converting phage Stx2_EH1910	1	LC567834.1
Enterobacteria phage PPompW_EH2201	1	LC567828.1
Enterobacteria phage PPompW_EH1992	1	LC567832.1
Enterobacteria phage PPompW_EH1846	1	LC567839.1
Enterobacteria phage PPompW_132418	1	LC567822.1
Enterobacteria phage PPompW_699	1	LC567826.1
Enterobacteria phage PPompW_EH2246	1	LC567836.1
Klebsiella phage ST13-OXA48phi12.3	1	MK422451.1
Nostoc phage A1	1	KU234533.1

Salinivibrio virus SMHB1	1	KX774374.1
Salinivibrio virus SMHB1	1	NC_047775.1
Salmonella phage 146851_sal5	1	KU927491.1
Salmonella phage 103203_sal4	1	KU927495.1
Salmonella phage 118970_sal4	1	KU878967.1
Salmonella phage 118970_sal4	1	NC_030919.1
Salmonella phage 146851_sal4	1	KU927492.1
Salmonella phage 64795_sal4	1	KU927498.1
Salmonella phage 101962B_sal5	1	KU927496.1
Salmonella phage 103203_sal5	1	KU927494.1
Salmonella phage 103203_sal5	1	NC_031946.1
Salmonella phage SPN9CC	1	NC_017985.1
Salmonella phage SPN9CC	1	JF900176.1
Caudovirales sp.	1	MH622910.1
Salmonella virus SPN1S	1	KC911856.1
Salmonella phage SPN9TCW	1	JQ691610.1
Salmonella virus SPN1S	1	KC911857.1
Salmonella virus SPN1S	1	NC_016761.1
Salmonella virus SPN1S	1	JN391180.1

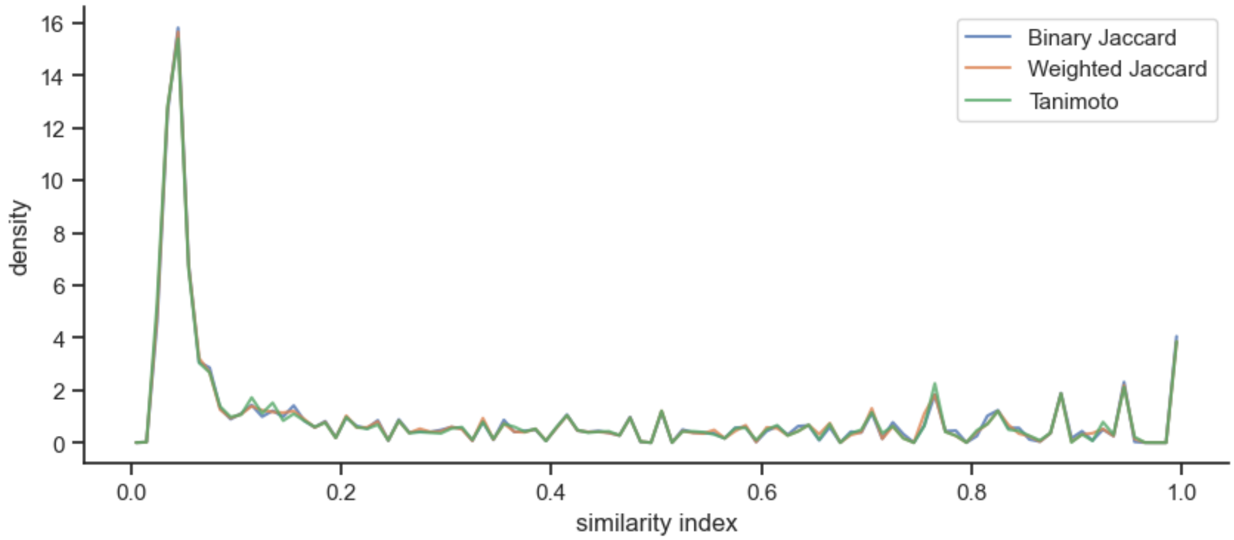

Figure S1. Comparison of the distribution of three similarity indices considered for this study. The distributions only marginally differ and the binary Jaccard was selected for further analyses.

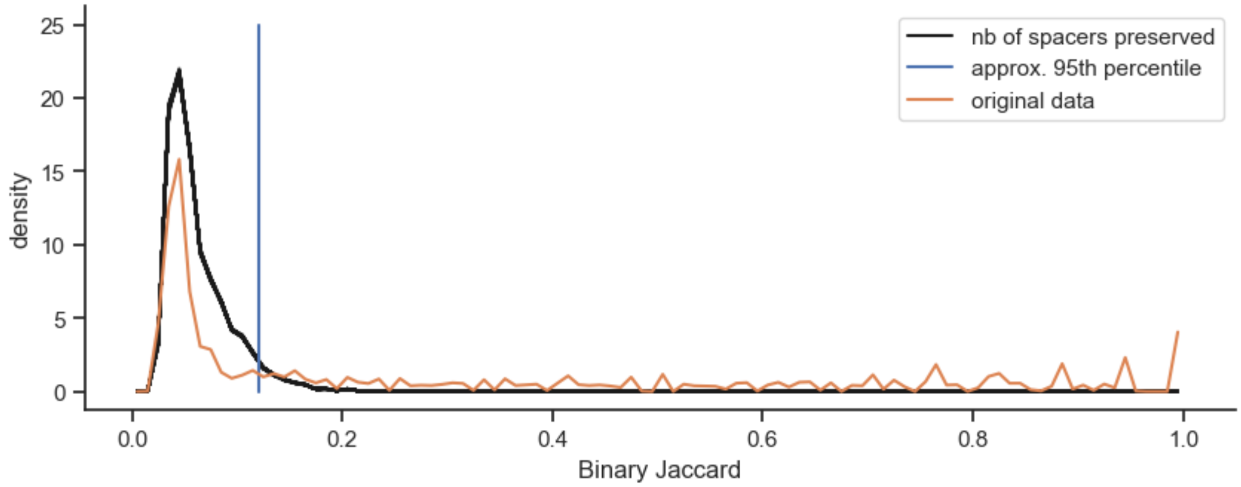

Figure S2. Comparison of the distribution of the value of the similarity indices in 500 random datasets with that of the original dataset. The 95th percentile is estimated at a Binary Jaccard value of 0.12.
